# Primary renal magnesium wasting: an unusual clinical picture of exercise‐induced symptoms

**DOI:** 10.14814/phy2.12773

**Published:** 2016-04-25

**Authors:** Christopher M. Stark, Cade M. Nylund, Gregory H. Gorman, Brent L. Lechner

**Affiliations:** ^1^ Department of PediatricsWalter Reed National Military Medical CenterBethesdaMaryland; ^2^ Department of PediatricsUniformed Services University of the Health SciencesBethesdaMaryland

**Keywords:** Adolescent, exercise‐induced, gastrointestinal, hypermagnesuria, hypomagnesemia, tetany

## Abstract

Magnesium is one of the most abundant cations in the human body and plays a key role as a metabolic enzyme cofactor and regulatory ion for neurons and cardiomyocytes. Hypomagnesemia due to isolated primary renal magnesium wasting is a rare clinical condition typically associated with neurological hyperexcitability. Exercise‐related gastrointestinal symptoms are caused by ischemic, mechanical, or neurohormonal changes. The role of hypomagnesemia in gastrointestinal symptoms is not well understood. We present a case of a 15‐year‐old male who presented with exercise‐induced abdominal pain, nausea, and vomiting, who was found to have profound hypomagnesemia and inappropriately elevated fractional excretion of magnesium (FEMg). Testing for multiple intrinsic and extrinsic etiologies of renal magnesium wasting was inconclusive. He was diagnosed with primary renal magnesium wasting and his symptoms resolved acutely with intravenous magnesium sulfate and with long‐term oral magnesium chloride. Primary renal magnesium wasting is a rare clinical entity that can cause extreme hypomagnesemia. It has not been associated previously with exercise‐induced gastrointestinal symptoms. The effects of hypomagnesemia on the human gastrointestinal tract are not well established. This case offers unique insights into the importance of magnesium homeostasis in the gastrointestinal tract. Exercise‐induced splanchnic hypoperfusion may contribute to gastrointestinal symptoms observed in this chronically hypomagnesemic patient.

## Introduction

Magnesium is vital for muscle electrolyte homeostasis, oxygen uptake, and energy production (Swaminathan 2003). Diseases of renal magnesium wasting, such as Gitelman syndrome, Bartter syndrome, and familial hypomagnesemia with hypercalciuria and nephrocalcinosis, are generally rare genetic disorders typified by specific gene mutations, protein defects, and inheritance patterns (Naderi and Reilly 2008). Individual diseases have characteristic serum and urine electrolyte findings that are influenced by specific protein function abnormalities. Diagnosis is ultimately made through laboratory or genetic testing. Primary renal magnesium wasting is a rare cause of hypomagnesemia that is characterized by hypermagnesuria.

Magnesium balance diseases are typically diagnosed with symptom onset in early childhood or adolescence. Hypomagnesemia symptoms are often difficult to isolate because they are relatively nonspecific and are frequently accompanied by other electrolyte imbalances (Table**** 1) (Pham et al. 2014; Konrad and Schlingmann 2014). Additionally, symptom onset may be indolent and nonspecific at initial presentation (Pham et al. 2014). Hyperexcitable neuromuscular manifestations are common, including tetany, seizures, and involuntary movements. Cardiac electrophysiologic effects, including lengthened QTc intervals, are often present, due to the role of magnesium in myocardial ion fluxes. Magnesium deficiency symptoms are typically not observed until serum levels decrease below 1.2 mg/dL (0.5 mmol/L). The rate of magnesium deficiency development and total body deficit may be more determinant for symptom onset than serum magnesium concentration (Swaminathan 2003). Hypomagnesemia can occur through multiple mechanisms, including decreased dietary intake and impaired magnesium absorption, secretion, or utilization **(**Table**** 2 **)**(Pham et al. 2014; Konrad and Schlingmann 2014; Jahnen‐Dechent and Ketteler 2012).

**Table 1:** Clinical manifestations of hypomagnesemia

Neurologic	Depression, delirium, coma, vertigo, nystagmus
Neuromuscular	Tetany, muscle cramps, seizures, involuntary movements
Cardiovascular	Widening QRS complex, prolonged PR or QT interval, ventricular arrhythmias, torsades de pointes
Electrolyte disturbances	Hypokalemia, hypocalcemia

**Table 2:** Common mechanisms of hypomagnesemia

Source of Hypomagnesemia	Mechanism	Potential Etiologies
Gastrointestinal		
Passive absorption (small intestine)	Decreased electrochemical gradient and bulk transport	Low dietary magnesium intake, short‐gut syndrome, proton pump inhibitors
Active absorption (large intestine)	Impaired transporter function	TRPM6 protein defects
Renal		
Glomerular filtration	Hyperfiltration leading to overwhelming of renal magnesium reabsorption capacity	Diabetes mellitus, acute tubular necrosis diuresis, excessive volume expansion
Proximal tubular reabsorption	Nephron cellular injury impairing passive reabsorption	Fanconi's syndrome, drug toxicity (aminoglycosides, cisplatin)
Thick ascending limb of Loop of Henle	Acquired or inherited impaired transporter function	Claudin‐16, Claudin‐19, NKCC2, ROMK, ClC‐Kb, or CaSR protein defects
Distal convoluted tubule	Acquired or inherited impaired transporter function	TRPM6, Kv1.1, Kir4.1/Kir5.1, NCCT, FXYD2, HNF1B, PCBD1, EGF, CaSR, or Cyclin M2 protein defects

TRPM6, transient receptor potential melastatin 6 (*TRPM6*); NKCC2, sodium–potassium‐chloride transporter 2 (*SLC12A1*); ROMK, renal outer‐medullary potassium channel (*KCNJ1*), ClC‐Kb, chloride channel Kb (*CLCNKB*); CaSR, calcium‐sensing receptor (*CASR*); Kv1.1, potassium voltage‐gated channel subfamily A member 1 (*KCNA1*); Kir4.1/Kir5.1, inward rectifier‐type potassium channel 4.1/5.1 (*KCNJ10*/*KCNJ16*); NCCT, sodium‐chloride symporter (*SLC12A3*); FXYD2, sodium‐potassium ATPase subunit gamma (*FXYD2*); HNF1B, hepatocyte nuclear factor‐1 beta (*HNF1B*); EGF, epidermal growth factor (*EGF*); PCBD1, pterin‐4‐alpha‐carbinolamine dehydratase 1(*PCBD1*); claudin‐16 (*CLDN16*); claudin‐19 (*CLDN19*); cyclin M2 (*CNNM2*).

Exercise‐induced gastrointestinal symptoms are relatively common among athletes and are provoked through ischemic, mechanical, or neuroendocrine mechanisms (de Oliveira and Burini 2009). Strenuous exercise causes blood shunting away from visceral organs, toward the skeletal muscle, lungs, heart, and brain. This physiologic blood redistribution, along with significantly decreased splanchnic blood flow, may cause localized ischemic damage, which is subsequently worsened by dehydration (Qamar and Read 1987). Patients with exercise‐induced symptoms frequently experience nausea, vomiting, abdominal cramping, diarrhea, and constipation (Casey et al. 2005). Body magnesium stores are redistributed during exercise to accommodate changing metabolic needs (Nielsen and Lukaski 2006). Previous studies suggest that hypomagnesemia has a detrimental effect on exercise performance and amplifies the negative results of strenuous exercise (Nielsen and Lukaski 2006).

We present an adolescent male with hypomagnesemia and hypermagnesuria caused by primary renal magnesium wasting. He experienced chronic, recurrent exercise‐induced tetany, abdominal pain, nausea, and vomiting. He was found to have persistent, inappropriately increased renal magnesium excretion in the absence of known causes, and required continuous magnesium replacement to avoid symptoms. Our review of the literature identified no other cases of exercise‐induced gastrointestinal symptoms related to hypomagnesemia and primary renal magnesium wasting.

## Case

A 15‐year‐old African American male was referred for pediatric gastroenterology evaluation with over 1 year of worsening recurrent exercise‐induced abdominal pain, nausea, and vomiting. He was an active athlete and reported that, exclusively during periods of strenuous physical exertion, he would develop severe multifocal abdominal pain. His pain was followed by nausea and emesis, which would persist through the day. He previously sought treatment and had no symptomatic improvement with ranitidine, omeprazole, and aggressive hydration with physical activity. One year prior to presentation, he was prescribed once daily omeprazole for presumed gastritis. He reported intermittent compliance throughout the year with this medication regimen, but relied primarily on activity reduction for symptom management. No laboratory studies were evaluated prior to this medication prescription. One month prior to presentation, his primary care provider drew a complete blood cell count, basic metabolic panel, and iron studies, which revealed no electrolyte disturbances and a mild microcytic anemia; so, ferrous sulfate supplementation was initiated and omeprazole was represcribed. No magnesium studies were evaluated at that time. At the time of presentation, his review of systems was positive for headaches, sometimes associated with emesis, fatigue, night sweats, and constipation. He denied any muscle cramps, tetany, light‐headedness, syncope, palpitations, convulsions, diarrhea, hematochezia, or weight loss. He denied any recent tobacco, alcohol, or drug use. His parents denied any family history of neurological diseases, myopathies, metabolic diseases, or genetic syndromes.

Physical exam was significant for an elevated blood pressure of 138/83 mm Hg and a rectal exam with a large hard stool in the rectum and negative fecal occult blood test. An abdominal film showed mild stool retention. A metabolic panel with magnesium was ordered and the patient was released home with instructions for treatment of constipation and close follow‐up with test results. Later that day, the provider received laboratory notification of a critically low serum magnesium level of 0.7 mg/dL (normal range 1.6–2.3 mg/dL) and otherwise normal metabolic panel. The patient returned for repeat serum magnesium measurement, which was 0.8 mg/dL. An electrocardiogram showed normal rate and normal sinus rhythm, with mild T‐wave flattening and no QT interval prolongation. The patient denied any active symptoms, but was admitted for magnesium normalization and further testing.

Upon admission, he had continued mild hypertension. A focused neurological exam was negative for tetany. Laboratory tests while hospitalized revealed a mild hypokalemia and slightly elevated serum bicarbonate, which resolved with magnesium replacement. Urinary electrolytes prior to magnesium repletion found an inappropriately elevated fractional excretion of magnesium (FEMg) at 7.8% (normal range = 0.5–4%). His fractional excretions of potassium, sodium, and chloride were within age‐appropriate reference ranges. Plasma parathyroid hormone and urine protein, calcium, phosphorus, creatinine, sodium, potassium, and chloride were normal. Thyroid function tests, transaminase levels, lipase, plasma renin activity, aldosterone, urine catecholamines, urinalysis, and urine culture were normal. Heavy metal, lead, and urine screen for diuretics were negative.

While hospitalized, he received 2 g of intravenous magnesium sulfate with subsequent serum magnesium level improvement. Oral magnesium oxide supplementation was initiated at 40 mg/kg/day. He was observed on telemetry and his ECG normalized after magnesium replacement. He remained asymptomatic throughout his hospitalization and maintained serum magnesium in the range of 1.0–1.2 mg/dL. He was discharged with instructions to continue his current dose of oral magnesium and avoid strenuous activity until follow‐up. Three days later, his serum magnesium remained above 1.2 mg/dL and his ECG remained normal, so he was allowed to continue activity with instructions to stop and seek treatment if symptoms returned. An outpatient renal ultrasound and upper gastrointestinal endoscopy series revealed no evidence of disease. An acylcarnitine profile analysis revealed no evidence of disorders of fatty acid oxidation or organic acid metabolism. Genetic testing for known mutations associated with Gitelman syndrome was negative. Results of genetic testing for additional disorders of renal magnesium loss were unavailable.

With frequent follow‐up, the patient's magnesium level remained borderline low (1.1–1.3 mg/dL) until 8 months after his initial discharge. Routine serum magnesium monitoring at that time showed a gradual decrease and the patient admitted to declining compliance with oral supplementation. Additionally, his exercise‐induced symptoms gradually had returned. He began to receive supplementary 1 g intramuscular magnesium sulfate injections with clinic appointments, in addition to continued oral magnesium, to help augment missed doses. His magnesium level at a subsequent follow‐up was noted to be 0.7 mg/dL with a FEMg elevated at 11%. He acknowledged continued poor oral adherence and significant recurrence of exercise‐induced symptoms. He underwent a graded exercise test, which revealed a slightly prolonged QTc of 447, and induced lower extremity cramping and complete reproduction of his abdominal pain, nausea, and vomiting. He was once again admitted for intravenous magnesium replacement. After repeated magnesium replacement, he was discharged with a simplified regiment of oral magnesium chloride, and subsequently had improved compliance.

## Discussion

The association between hypomagnesemia and exercise‐induced gastrointestinal symptoms could be from exercise‐induced, acute‐on‐chronic hypomagnesemia, causing decreases in bowel motility and shifts in splanchnic circulation. Magnesium plays an essential role in smooth muscle kinase activity, but our review of the literature found no human in vitro or in vivo studies of hypomagnesemia and intestinal function (Webb 2003). Magnesium‐deficient sheep were found to have decreased contraction frequency and amplitude of the cecum and proximal colon, and an absence of normal increase in postprandial reticulo‐rumen contraction frequency (Bueno et al. 1980). Hypomagnesemia contributed to a significant reduction in the duration of spiking activity in the spiral (ascending) colon. The amplitude of small intestine regular spiking activity and number of daily migrating myoelectric complexes (MMC) also were decreased. One case series found magnesium deficiency in seven of 11 patients with intestinal pseudo‐obstruction (IPO), a rare intestinal motility impairment syndrome that causes pain, nausea, and vomiting (Hirsh et al. 1981). These patients reported symptomatic relief after their magnesium levels were normalized. Another report describes a hypomagnesemic woman with diffuse esophageal smooth muscle spasm (DES) that recovered following magnesium supplementation (Iannello et al. 1998). The role of magnesium balance in IPO and DES pathophysiology has not been studied previously.

Exercise‐induced gastrointestinal symptoms are caused by shifts in blood flow and electrolyte balances in response to changing metabolic demands. During physical activity, body magnesium stores shift transiently from the plasma into skeletal muscle and adipose tissue to increase energy production and counteract oxidative stress (Nielsen and Lukaski 2006). Magnesium fluxes in hypomagnesemic patients are poorly understood, but studies suggest that compartmental shifting may be altered (Westmoreland et al. 2004). Splanchnic hypoperfusion occurs, to some degree, in all healthy individuals during exercise, and can cause transient dysfunction of intestinal cellular membranes (van Wijck et al. 2011). When splanchnic blood flow is decreased, erythrocyte magnesium availability and local plasma volume are reduced (Qamar and Read 1987).

Although this patient's unique clinical presentation offers insight into the importance of magnesium in gastrointestinal smooth muscle homeostasis, his extremity tetany suggests that physiologic blood shunting is not the sole mechanism of his symptoms. In the presence of increased blood flow, hypomagnesemic patients may fail to meet the threshold to prevent skeletal muscle hyperexcitation symptoms normally ameliorated by exercise‐induced, transient magnesium fluxes. One clinical report described an otherwise healthy young female athlete with recurrent episodes of exercise‐induced muscle spasms, who was found to be profoundly hypomagnesemic (Liu et al. 1983). Her symptoms resolved with magnesium replacement. Normomagnesemic patients may avoid the symptom threshold with body magnesium stores, but hypomagnesemic patients can be overwhelmed during strenuous exercise. Concurrent increases of urine and sweat magnesium losses during exercise likely contribute to exercise‐induced symptoms (Nielsen and Lukaski 2006).

Hypomagnesemia is commonly associated with other electrolyte abnormalities. Our patient was noted to have a mild hypokalemia and elevated serum bicarbonate during his hospitalization. The association between hypomagnesemia and hypokalemia is well known, as magnesium deficiency increases distal potassium secretion through decreased inhibition of the potassium‐secreting renal outer medullary potassium channels (Huang and Kuo 2007). Importantly, hypokalemia with concomitant hypomagnesemia is often refractory to management until magnesium levels have normalized. The patient's transient metabolic alkalosis resolved during his hospitalization and was likely related to mild hypovolemia at the time of admission. It is important to note that some medications, including proton pump inhibitors, are associated with decreased gastrointestinal magnesium absorption and clinically significant hypomagnesemia (Florentin and Elisaf 2012). Our patient had prolonged intermittent exposure to omeprazole, which may have augmented his hypomagnesemia, but this exposure is not typically associated with renal magnesium wasting. Further study is needed to explain the relationship between exercise and gastrointestinal magnesium homeostasis.

## Conflict of Interest

The authors declare that they have no conflict of interest. The views expressed in this article are those of the authors and do not reflect the official policy or position of the United States Army, the United States Air Force, the United States Navy, Department of Defense, or the U.S. Government.
